# LTBP2 regulates cisplatin resistance in GC cells via activation of the NF-κB2/BCL3 pathway

**DOI:** 10.1590/1678-4685-GMB-2023-0231

**Published:** 2024-04-05

**Authors:** Jun Wang, Wenjia Liang, Xiangwen Wang, Zhao Chen, Lei Jiang

**Affiliations:** 1 The First Hospital of Lanzhou University, Department of General Surgery, Ward 6, Lanzhou, Gansu, China.; 2 Gansu Provincial Hospital, Department of Ultrasound, Lanzhou, Gansu, China.

**Keywords:** Gastric cancer, cisplatin resistance, LTBP2, proliferation

## Abstract

Gastric cancer (GC) often develops resistance to cisplatin treatment, but while latent transforming growth factor β-binding protein (LTBP2) is recognized as a potential regulator in GC, its specific role in cisplatin resistance is not fully understood. This study investigated LTBP2’s impact on cisplatin resistance in GC. LTBP2 expression was assessed in various GC cell lines, and its correlation with cisplatin sensitivity was determined through cell viability assays. Lentivirus-mediated LTBP2 silencing in HGC-27 cells demonstrated enhanced cisplatin sensitivity, reduced cell proliferation, and inhibition of the NF-κB2/Bcl-3/cyclin D1 pathway. Additionally, transient transfection overexpressed the NFκB2 gene in LTBP2-silenced HGC-27/DDPR cells, restoring cisplatin sensitivity and upregulating p52/Bcl-3/cyclin D1. In conclusion, silencing LTBP2 could effectively inhibit cell proliferation and mitigate cisplatin resistance via the NFKB noncanonical pathway NFKB2 p52/Bcl-3/cyclin D1. These findings propose LTBP2 as a potential therapeutic target for overcoming cisplatin resistance in GC patients.

## Introduction

Gastric cancer (GC), a common gastrointestinal tumor, has a considerably high mortality rate. More than 1 million new diagnoses and approximately 769,000 deaths occurred in 2020. Surveys have found that GC incidence and mortality rates in China are higher than the world average (Sung et al., 2021). With the development of surgical techniques, the prognosis of patients with early gastric cancer has improved. However, when diagnosed, most patients already have advanced GC, and chemotherapy has become one of the main treatment strategies (Chandra et al., 2021). Cisplatin-based chemotherapy remains the mainstay of GC treatment. Nevertheless, to a large extent, the efficacy of cisplatin is limited by drug resistance, leading to lower survival rates (Zheng et al., 2017). Therefore, finding new solutions to enhance cisplatin sensitivity is vital.

Latent transforming growth factor-β-binding protein (LTBP2), an extracellular matrix (ECM) protein is affiliated to the fibrillin/LTBP ECM glycoprotein family. A study suggested that poor survival in GC patients is associated with LTBP2. Silencing *LTBP2* effectively inhibits GC cell migration, proliferation, invasion, and epithelial-mesenchymal transition (Wang et al., 2018). Wang *et al*. (2022) indicated that knocking down LTBP2 can activate the p62-Keap1-Nrf2 pathway to promote ferroptosis in GC cells, thereby inhibiting GC progression. The studies above suggest that *LTBP2* affects the cellular functionalities of GC, but its role and mechanism in chemoresistance are unclear. This study focused on the function of *LTBP2* in chemotherapy resistance and how *LTBP2* affects cisplatin resistance in GC cells. By analyzing GEO shared data (GSE191323 and GSE186205), we suggested that silencing *LTBP2* might improve GC cisplatin sensitivity by inhibiting NF-κB2.

The nuclear factor-κB (NF-κB) pathway is divided into 1. the classical group, which is usually activated by dimerization of RelA and NF-κB1, and 2. the alternative group, which is activated by RelB-NF-κB2 dimerization. NF-κB is associated with the inflammatory response and is synthesized as a 100-kDa precursor protein (NF-κB2 p100) (Smale, 2012). The p100 protein is, in turn, cleaved by the proteasome to produce the functional molecule p52. Activation of the NF-κB2/p52 pathway is thought to be a causal factor in gastric carcinogenesis (Burkitt et al., 2013). B-cell lymphoma 3 (Bcl-3), a member of the NF-κB protein family, is associated with the subcellular translocation of NF-κB and DNA binding (Legge et al., 2020). Bcl-3 can promote cell migration and chemoresistance in GC cells (Hu et al., 2020). p52 forms a trimeric complex with Bcl3 that mediates gene transcription related to the promotion of tumor cell proliferation, survival, invasion, and metastasis (Wu et al., 2018).

In summary, in this study, it was tentatively hypothesized that LTBP2 might activate the unconventional NF-κB pathway by regulating *NF-κB2* gene expression, thereby inducing cisplatin resistance in GC cells, and further experiments were conducted to demonstrate this in combination with the HGC-27 cisplatin-resistant cell line.

## Material and Methods

### Bioinformatics research

“Cisplatin resistance in GC” and “*LTBP2* silencing” were queried in the GEO database (https://www.ncbi.nlm.nih.gov/geo/) to obtain details of relevant differentially expressed genes. The GEO database (https://www.ncbi.nlm.nih.gov/geo/) retrieval of “gastric carcinoma cisplatin resistance” and “LTBP2 silencing” related terms and the corresponding gene differentially expressed detail databases “GSE186205” (there were six human samples in total, three of which were signet ring KATOIII gastric cancer cells and the other three were cisplatin resistance KATO/DDP cell lines) and “GSE191323” (six human samples, three fibroblasts and three LTBP2 silenced fibroblasts) were analyzed. A total of 483 genes were significantly upregulated after cisplatin treatment in the GSE186205 data, and 2614 genes were downregulated considerably after LTBP2 silencing in the GSE191323 data. A total of 62 genes were selected, in combination with the Kaplan-Meier-Meier plotter (http://kmplot.com/analysis/) (Győrffy, 2023), to analyze the prognosis of gastric adenocarcinoma demonstrating mitoses. High gene expression was selected, and the prognosis was poor (19 genes, P < 0.01). GEPIA (http://gepia.cancer-pku.cn/) to analyze the correlation between the above 19 genes and the *LTBP2* gene (P<0.01). Focusing on genes involved in the regulation of gene transcription, NFκB2 was finally identified as the research target.

### Cell culture

HGC-27 cells and HGC-27/DDPR (HGC-27 cisplatin-resistant strain) cells were obtained from Zhejiang Ruyao Biotechnology Co., LTD. RPMI-1640 medium containing 20% fetal bovine serum + 1% penicillin/streptomycin was used to culture cells. For HGC-27/DDPR cells, an additional 1 μM cisplatin (purity: 99.7%; CAS No. 15663-27-1, MedChemExpress USA) was added to the medium for maintenance. Cells were incubated at 37 °C with 5% CO_2_ in a constant temperature incubator.

### qRT‒PCR

GES-1 (normal gastric epithelial cells) and AGS, MKN-74, MKN-45, MKN-7, HGC-27 (GC cell lines), and HGC-27/DDPR cells were obtained from Zhejiang Ruyao Biological Co., LTD. For HGC-27 and HGC-27/DDPR cells, mRNA was extracted using the TRIzol method after different treatments. mRNA reverse transcription was performed using the 1st Strand cDNA Synthesis Kit gDNA Purge kit (Novoprotein, China). qPCR was performed under the following conditions: 2 min at 95 °C followed by 40 cycles of 95 °C for 10 s, 60 °C for 10 s and 72 °C for 30 s. The 2^-ΔΔCt^ method was used to calculate the relative expression of target genes (Rao et al., 2013). The amplification primers were as follows: LTBP2 Forward: 5’- CTG CAC AGA TGA CAA CGA GTG TC-3’, Reverse: 5’- AGA GTG TAG CCA GGG TAG CAG A-3’; NF-κB2 Forward: 5’- GGC AGA CCA GTG TCA TTG AGC A-3’, Reverse: 5’- CAG CAG AAA GCT CAC CAC ACT C-3’; β-actin Forward: 5’- GGC AGA CCA GTG TCA TTG AGC A-3’, Reverse: 5’- CAG CAG AAA GCT CAC CAC ACT C-3’ (designed by the NCBI online website).

### Western blot

Total protein was extracted from HGC-27 and HGC-27/DDPR GC cells after RIPA (Boster, China, AR0102) lysis. A BCA assay kit was used to determine the protein concentration. Twenty micrograms of protein were denatured, separated by electrophoresis, and then transferred to PVDF membranes (Millipore, USA). The membranes were blocked at room temperature for 1 h with 5% skim milk and then mixed with primary antibodies overnight at 4 °C. The secondary antibodies, goat anti-rabbit or mouse IgG-HRP, were coincubated at room temperature for 1 hour the next day. Protein strips were developed and photographed by exposure using a chemiluminescence substrate kit (Boster, China) and a UVP gel UV imager (ChemiDoc-It Imaging System, USA). The optical density values of each strip were quantified by ImageJ software.

Primary antibodies against LTBP2 (sc-166199, Santa Cruz, USA), P-gp (ET1611-30, Huabio, China), Bax (ET1603-34, Huabio), Bcl-2 (ET1610-20, Huabio), cleaved caspase-3 (ET1602-47, Huabio), cyclin D1 (ET1601-31, Huabio), Bcl-xL (ET1603-28, Huabio), Bcl-3 (ab259832, Abcam, USA), NFκB2 (EM1901-78, Huabio) and β-actin (ET1701-80, Huabio)

### CCK-8 assay for cellular activity

GC cell viability was assayed using Cell Counting Kit 8 (CCK-8) (cat no. CK04, Dojindo, China). HGC27 and HGC27/DDP cells were cultured at a 5000 cells/well density in 96-well plates. After 24 h, 5 μM cisplatin was administered and incubated for 0, 24, 48, and 72 h. After the medium was removed, 100 μL of 10% CCK-8 solution was added to each well and incubated in the dark for 2 h at 37 °C. A microplate reader (Thermo Fisher Scientific, Vantaa, Finland) was used to measure the absorbance at 450 nm.

### Construction of LTBP2-silenced cell lines

For the LTBP2 CDS region sequence, LTBP2 shRNA was designed using the GPP Web Portal online website (https://portals.broadinstitute.org/gpp/public). GENEWIZ (Suzhou, China) was commissioned to synthesize the complementary single-stranded oligonucleotides containing the target sequences. The annealed double-stranded sequences were as follows: shRNA1: Forward: 5’- CCG GAG TCT GGC TTC CGC ATC TAT TCT CGA GAA TAG ATG CGG AAG CCA GAC TTT TTT G-3’, Reverse: 5’- AAT TCA AAA AAG TCT GGC TTC CGC ATC TAT TCT CGA GAA TAG ATG CGG AAG CCA GAC T-3’; shRNA2: Forward: 5’- CCG GGT CTG GCT TCC GCA TCT ATT TCT CGA GAA ATA GAT GCG GAA GCC AGA CTT TTT G -3’, Reverse: 5’- AAT TCA AAA AGT CTG GCT TCC GCA TCT ATT TCT CGA GAA ATA GAT GCG GAA GCC AGA C-3’; shRNA3: Forward: 5’- CCG GTC TGG CTT CCG CAT CTA TTT CCT CGA GGA AAT AGA TGC GGA AGC CAG ATT TTT G-3’, Reverse: 5’- AAT TCA AAA ATC TGG CTT CCG CAT CTA TTT CCT CGA GGA AAT AGA TGC GGA AGC CAG A-3’. Oligonucleotides were linked to the pLKO.1-puro plasmid after double digestion with *Age*I and *Eco*RI and then transformed into Stabl3 receptor cells. The shRNA inserts were confirmed using DNA testing, and the Endotoxin-free Plasmid DNA Bulk Kit (cat. no. 1017025, Semgen, China) was used for plasmid extraction.

Lentivirus preparation was performed using the cotransfection method. The recombinant plasmid, △8.91 and pVSV-G (mass ratio 10:10:1) were transfected into 293T cells using the cationic lipid complex method (X-tremeGENE HP DNA transfection reagent, Roche). The cell supernatant was collected after 48 h, and the target cells were infected after passing through a 0.45 μm filter. Cells were divided into 4 groups: shScramble group and LTBP2 shRNA 1-3 group. Viral supernatants from different groups were added to HGC27 and HGC27/DDP cells with 2 µg/ml polybrene to promote infection. Forty-eight hours later, fluorescence was confirmed. Afterwards, the original medium was replaced with a complete medium containing 2 μg/ml puromycin, and the cells were cultured for 7-9 days. Western blotting was used to determine the protein expression levels of LTBP2.

### Effect of silencing LTBP2 on cell activity and proliferation

The activity of HGC-27 and HGC-27/DDPR cells at 0, 24, 48, and 72 h of *LTBP2* silencing was detected by referring to the CCK-8 instructions. The proliferation of LTBP2-silenced HGC-27 and HGC-27/DDPR cells was detected by combining the BeyoClick™ EdU-594 Cell Proliferation Assay Kit (C0078S, Beyotime, China) and fluorescence microscopy (DM500, Leica), and the positivity rate was calculated.

In addition, LTBP2-silenced HGC-27 and HGC-27/DDPR cells were inoculated at 300 cells/well into six-well plates. Two millilitres of complete medium was added to each well. Cells were incubated in a constant temperature incubator containing 5% CO_2_ at 37 °C until the cell clones could be directly observed. The medium was removed, and methanol was added to fix the cells for 30 minutes. The methanol was removed, and the cells were stained with crystal violet for 30 minutes and counted.

### Immunofluorescence

LTBP2-silenced HGC-27 and HGC-27/DDPR cells were wall-cultured on 6-well plates containing coverslips. Immunofluorescence staining was performed after 48 h. Treated cells were incubated overnight with primary antibodies (anti-LTBP2 and NF-κB2) at 4 °C. The secondary antibody was goat anti-rabbit or mouse IgG-FITC, and the cells were coincubated for 1 h. The cells were observed and photographed using fluorescence microscopy after staining the nuclei with 1 μg/mL DAPI for 15 minutes. This process included the analysis of three distinct fields of view to ensure comprehensive cellular assessment. IPP6.0 statistically quantified the results.

### Construction of NF-κB2-overexpressing cells

Coding sequences (CDSs) of NF-κB2 were sourced from NCBI. The high-fidelity enzyme KOD was utilized for amplifying the CDS. For vector construction, pCDH plasmid was employed. The plasmid and the amplified NF-κB2 product were doubly cleaved using the restriction endonucleases *Xba*I and *Bam*HI, followed by ligation with T4 DNA ligase. Gene sequencing confirmed the successful construction of the recombinant plasmid. NF-κB2 amplification primers: *Xba*I-Forward: 5’-GCT CTA GAC CCA GAG ACA TGG AGA GTT GCT, *Bam*HI-Forward: 5’- CGG GAT CCA GCA GGT CAG TGC ACC TGA. LTBP2-silenced HGC-27 and HGC-27/DDPR cells were cultured in 6-well plates at 37 °C at 1×10^6^ cells/well. When the cell confluency was 60%-70%, 5 μg of pCDH-NF-κB2 and pCDH negative plasmid (pCDH-NC) were then transfected into GC cells by X-tremeGENE HP DNA transfection reagent. qPCR was used to measure the transfection efficiency after 48 h of transfection.

### Effect of overexpression of *NF-*κ*B2* and silencing of *LTBP2* on GC cells

HGC-27 and HGC-27/DDPR cells were divided into 4 groups: 1, scramble group; 2, sh-LTBP2 group; 3, sh-LTBP2+OE-NC group, silencing *LTBP2* with pCDH-NC infection; and 4, sh-LTBP2+OE-NF-κB2 group, silencing *LTBP2* with pCDH-NF-κB2 infection. The cellular activity levels of the above-grouped cells were assayed by CCK-8 assay (all under 5 μM cisplatin pressure). Then, the expression levels of LTBP2/NF-κB2 proteins and the downstream regulation of NF-κB2 proteins and transcriptional proteins Bcl-3/Bcl-xL/Cyclin D1 proteins were measured by Western blot (Zhang et al., 2007).

### Statistical analysis

Three independent replicates were examined in all cell experiments. GraphPad Prism software calculated data (mean ± SE of three replicates) (Prism version 8; GraphPad Software, Inc.). A *t*-test was used to compare the two groups. P<0.05 indicates a statistically significant difference.

## Results

### The correlation between *LTBP2* expression level and GC

**Figure 1 --:**
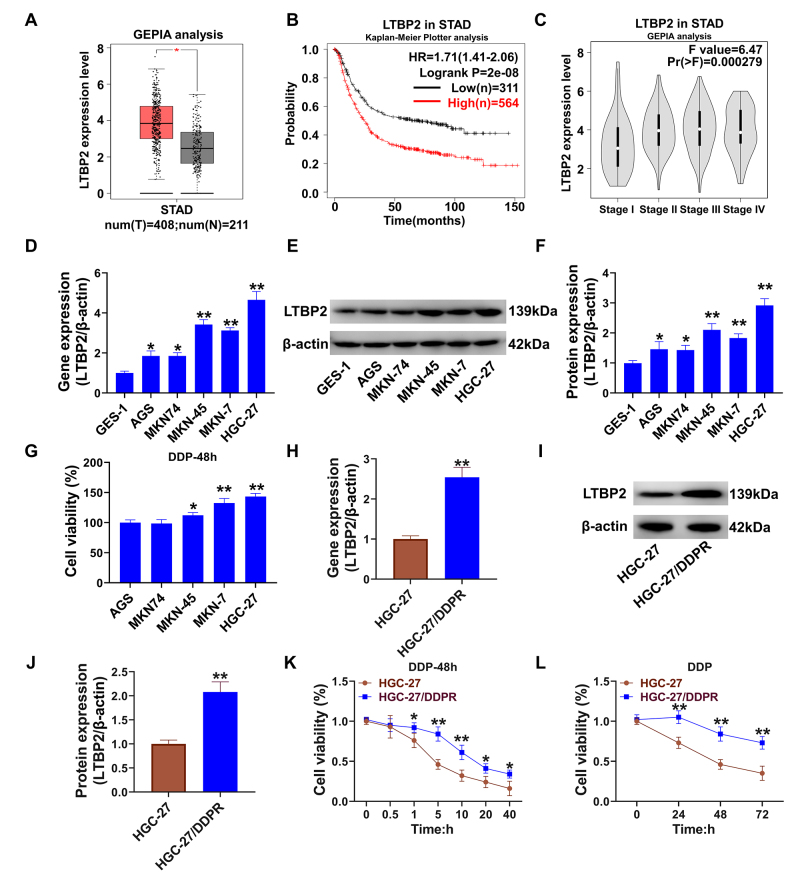
Correlation of *LTBP2* expression in GC patients and cell lines (A) Analysis of the differential expression levels of the *LTBP2* gene in gastric adenocarcinoma patients using the GEPIA online website; (B) Correlation between the *LTBP2* gene and prognosis of GC patients using Kaplan-Meier Plotter online website; (C) Analysis of the *LTBP2* gene expression level and GC disease stage using GEPIA online website; (D) qPCR to detect the relative expression level of *LTBP2* gene in different GC cell lines; (E) Western blot to detect the relative expression level of LTBP2 protein in different GC cell lines; (F) Quantitative analysis of the optical density of protein bands in western blot and statistical analysis; (G) CCK-8 assay to detect cell activity; (H) qPCR to detect the relative expression of *LTBP2* gene; (I-J) Quantification of the optical density of bands to evaluate LTBP2 protein expression; (K-L) CCK-8 assay for cellular activity. *P<0.05, **P<0.01, compared with the control group. Quantitative data are presented as means ± SD from three independent experiments.

To explore the research value of LTBP2 in gastric cancer, this part first analyzed the expression of the *LTBP2* gene in gastric adenocarcinoma (STAD) patients and healthy people’s gastric tissues through the GEPIA website. The *LTBP2* gene was significantly upregulated in STAD (P<0.05, Figure 1A). Kaplan‒Meier Plotter online website analysis of the *LTBP2* gene and the prognosis of gastric cancer patients showed that the prognosis of patients with high LTBP2 expression was significantly lower than that of patients with low *LTBP2* expression (P<0.01, Figure 1B). *LTBP2* gene expression levels rose with escalating tumor stage (Figure 1C). Among the GC cells tested, HGC-27 cells had the highest mRNA (Figure 1D) and protein (Figure 1E-F) expression levels (P<0.01, compared with GES-1). HGC-27 had the highest cellular activity after 5 μM cisplatin treatment for 48 h (Figure 1G). In cisplatin-resistant HGC-27/DDPR cells, *LTBP2* gene (Figure 1H) and protein (Figure 1I-J) levels were significantly higher than those in wild-type HGC-27 cells (P<0.01). When the concentration of cisplatin was greater than 5 μM and less than 10 μM, the difference in activity between HGC-27 wild-type and HGC-27/DDPR cells was extremely significant (P<0.01, Figure 1K). Therefore, we selected 5 μM as the working concentration. Treatment with 5 μM cisplatin has increased cellular activity in HGC-27/DDPR cells at 24, 48, and 72 h (P<0.01, Figure 1L).

### Effect of silencing *LTBP2* on the biological function of HGC-27 cells

**Figure 2 -:**
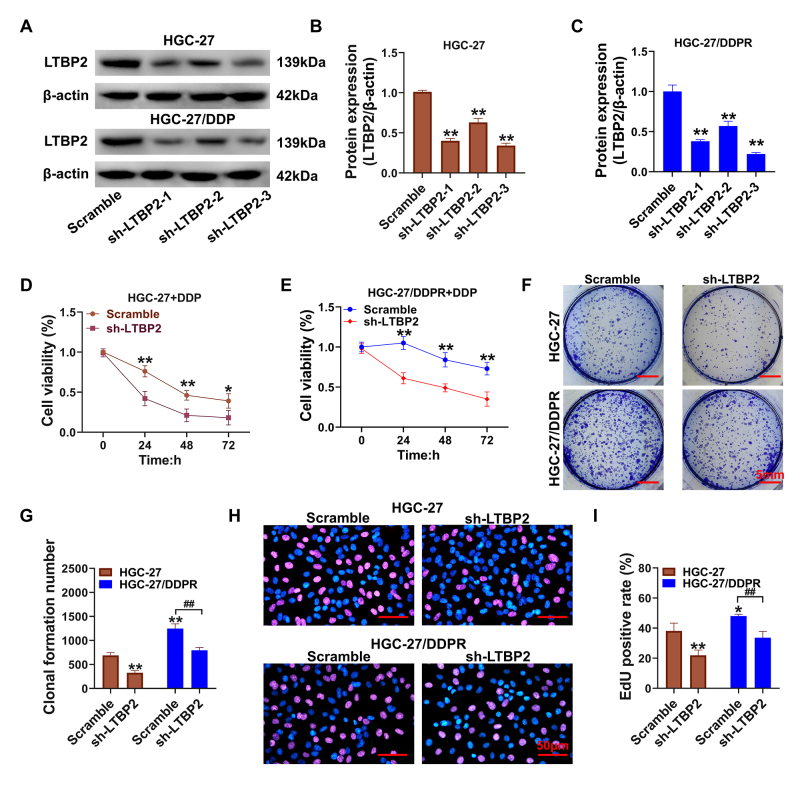
Effect of *LTBP2* silencing on cisplatin resistance and proliferative activity of HGC-27 cells (A) Assessment of LTBP2 silencing efficiency via western blot; (B-C) Quantitative analysis of the optical density values of the protein bands in western blot; (D-E) Evaluation of cisplatin sensitivity in GC cells following LTBP2 silencing using CCK-8 assay; (F-G) Quantitative analysis of clone formation, with a scale bar of 5mm; (H-I ) Assessment of cell proliferation levels using the EdU assay, with quantitative statistical analysis, and a scale bar of 50μm; **P<0.01, *P<0.05, compared with HGC-27 scramble group; ^##^P<0.01, ^#^P<0.05, linked groups for comparison. Quantitative data are presented as means ± SD from three independent experiments.

sh-LTBP2-3 showed the most significant inhibition of LTBP2 protein expression (P<0.01 compared with scramble Figure 2A-C). We also observed significantly increased sensitivity of wild-type HGC-27 and HGC-27/DDPR cells to 5 μM cisplatin after silencing *LTBP2* (Figure 2D-E, compared with the scramble group, P<0.01). Both colony formation (Figure 2F-G) and EdU assays (Figure 2H-I) showed that silencing *LTBP2* reduced survival, proliferation and the proportion of cells in a proliferative state. (P<0.01, sh-LTBP2 group compared to scramble group).

### Effect of silencing *LTBP2* on cisplatin-induced apoptosis in GC cells

**Figure 3 -:**
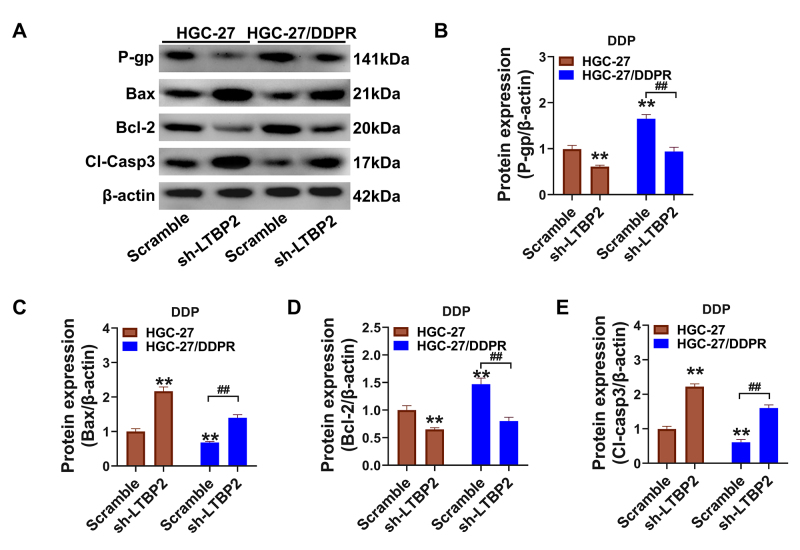
Effect of *LTBP2* silencing on the regulation of cisplatin-induced apoptosis levels in GC cells. (A) Expression levels of P-gp, Bcl-2, Bax, and cleaved caspase-3 assessed by western blot; (B-E) Quantification of the optical density values for the protein bands of P-gp, Bax, Bcl-2 and cleaved caspase-3. **P<0.01, *P<0.05, compared with HGC-27 scramble group; ^##^P<0.01, ^#^P<0.05, linked groups for comparison. Quantitative data are presented as means ± SD from three independent experiments.

We examined the levels of drug resistance-associated proteins and apoptosis to determine the variations in cisplatin resistance in HGC-27 cells with silenced LTBP2 (Figure 3A). The expression of P-gp, which is associated with drug resistance, and Bcl-2, which is related to anti-apoptosis, were significantly higher in HGC-27/DDPR cells than in HGC-27 cells, while the expression of Bax and cleaved caspase-3 were inhibited (Figure 3B-E, P<0.01), which are related to pro-apoptosis. In addition, the expression of P-gp and Bcl-2 was inhibited in wild-type HGC-27 and HGC-27/DDPR cells after silencing *LTBP2*. In addition, the expression of P-gp and Bcl-2 in wild-type HGC-27 and HGC-27/DDPR cells was suppressed. In contrast, the expression levels of Bax and cleaved caspase-3 were significantly upregulated after silencing LTBP2 (P<0.01, compared with the scramble group).

### Silencing *LTBP2* inhibits activation of the NF-KB2 p52/Bcl-3 pathway

**Figure 4 -:**
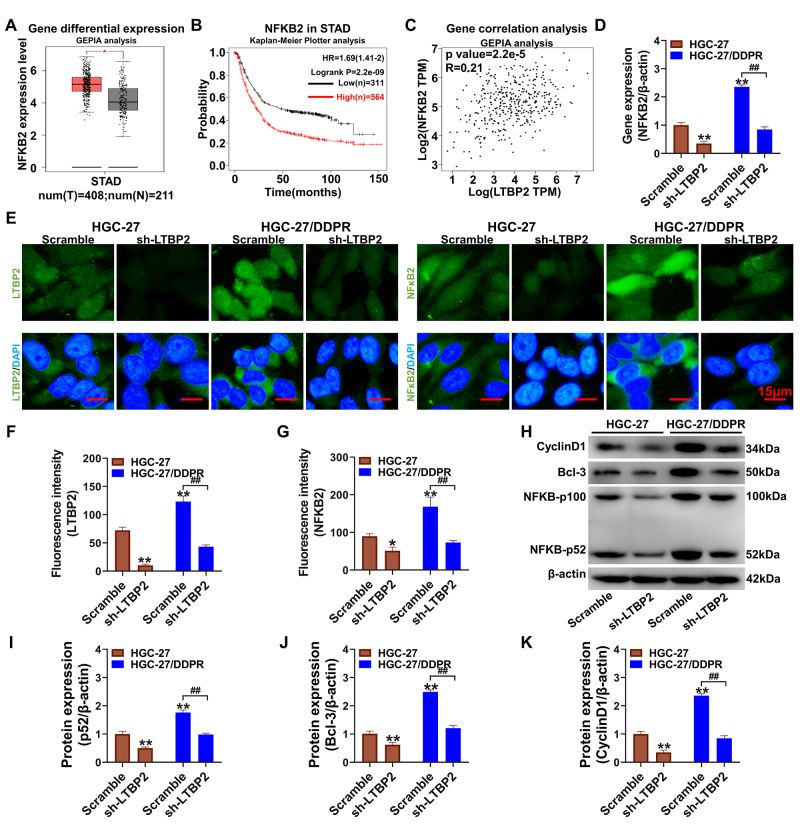
Silencing *LTBP2* can inhibit the activation of the NF-κB p52/Bcl-3 pathway (A) Analysis of the differential expression levels of NF-κB genes in gastric adenocarcinoma patients using the GEPIA online website; (B) Correlation analysis between NF-κB gene expression and the prognosis of GC patients using the Kaplan-Meier Plotter online website; (C) Assessment of the correlation between LTBP2 and NF-κB gene expression using the GEPIA online website; (D) Detection of NF-κB gene expression via qPCR; (E-G) Immunofluorescence to detect LTBP2 and NF-κB protein expression and distribution, the results were quantified by IPP6.0. Green fluorescence is the result of positive staining of the target protein, and blue fluorescence is DAPI staining of the nucleus. The scale bar is 15 μm. (H-L) western blot to detect NF-κB/ Bcl-3/ cyclin D1 protein expression and the results were quantified and counted. **P<0.01, *P<0.05, compared with HGC-27 scramble group; ^##^P<0.01, ^#^P<0.05, linked groups for comparison. Quantitative data are presented as means ± SD from three independent experiments.

We analyzed the genes in GSE186205 and GSE191323 regarding GC cisplatin resistance and differential regulation after silencing *LTBP2*. The results suggested that differential expression of NF-κB2 might be associated with LTBP2 induction of cisplatin resistance. The GEPIA website online analysis of NFKB2 gene differential expression levels showed that the NFKB2 gene was significantly upregulated in gastric adenocarcinoma tissues (Figure 4A, P<0.05). The Kaplan‒Meier plotter online website showed that gastric cancer patients with high NFKB2 expression had a poor prognosis, and the difference was statistically significant compared with patients with low NFKB2 expression (Figure 4B, P <0.01). The results of the GEPIA website analysis showed a positive correlation between *LTBP2* and *NFKB2* gene expression levels (R=0.21, P=2.2e-5, Figure 4C). After silencing *LTBP2*, the *NF-κB2* gene expression level was significantly downregulated in HGC-27 cells (P < 0.01, compared with that in the scramble group, Figure 4D), and the *NF-κB2* gene expression level was markedly higher in HGC-27/DDPR cells than in the wild-type HGC-27 cells (P < 0.01). In contrast, the expression of LTBP2 and NF-κB2 proteins was suppressed by silencing LTBP2 (P<0.01, compared with that in the scramble group, Figure 4E-G), and LTBP2 and NF-κB2 proteins were distributed in both the cytoplasm and nucleus. Western blotting further examined NF-κB2/Bcl-3 pathway proteins (Figure 4H-K), and the results suggested that the expression levels of NF-κB2-p52 protein and Bcl-3/Bcl-xL/cyclin D1 were significantly suppressed after silencing LTBP2 (P<0.01, compared with the scramble group).

### Overexpression of *NF-*κ*B2* reverses the effects of the NF-κB2 p52/Bcl-3 pathway by silencing *LTBP2*

**Figure 5 -:**
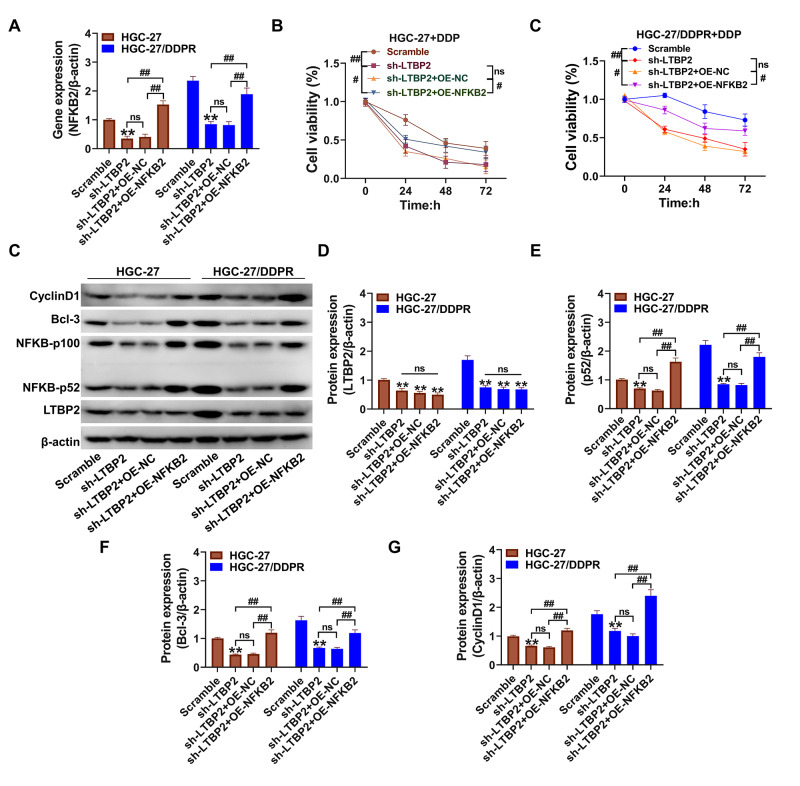
Overexpression of *NF-κB* can reverse the regulation of NF-κB p52/Bcl-3 pathway by silencing *LTBP2* (A) qPCR for *NF-κB* gene expression; (B-C) CCK-8 for cell activity; (D-H) Western blot for NF-κB/ Bcl-3/ cyclin D1 protein expression levels and the results Quantitative statistics were performed. **P<0.01, *P<0.05, compared with scramble group; ^##^P<0.01, ^#^P<0.05, linked groups for comparison. ns represents that the difference between the groups was not statistically significant. Quantitative data are presented as means ± SD from three independent experiments.

To investigate whether cisplatin resistance in GC cells is induced by LTBP2 through the regulation of NF-κB2, *NF-κB2* was overexpressed in this study for reversibility. qPCR results confirmed (Figure 5A) that *NF-κB2* gene expression levels were significantly upregulated after overexpression of NF-κB2 (P<0.01, compared with the sh-LTBP2 group). Meanwhile, as shown in Figure 5B,C, the activity of GC cells was also significantly upregulated after 20 μM DDP treatment (P<0.05, compared with the sh-LTBP2 group). Western blot results confirmed (Figure 5D-H) that overexpression of NF-κB2 significantly reversed the inhibitory effect of silencing *LTBP2* on p52/Bcl-3/Bcl-xL/cyclin D1 protein expression (P<0.01, sh-LTBP2+OE-NF-κB2 group compared with sh-LTBP2 group).

## Discussion

Chemoresistance is a major problem in the treatment of GC, and in particular, increased resistance to the first-line chemotherapeutic agent cisplatin usually leads to a poorer prognosis. The high expression of *LTBP2* also predicts poor prognosis outcomes (Wang et al., 2018). In this study, we performed transcriptomic analysis of cisplatin-resistant GC cells and *LTBP2* silencing using the GEO database (GSE186205 and GSE191323) and GEPIA. This result suggested that LTBP2 and NF-κB2 expression was significantly upregulated in cisplatin-resistant cell lines and that the *NF-κB2* gene was also considerably suppressed after *LTBP2* silencing. Therefore, we speculate that LTBP2 may regulate the expression of the *NF-κB2* gene and thus mediate cisplatin resistance.

To confirm the above speculation, we first examined the LTBP2 gene and protein expression in various GC cells. The results suggested that HGC-27 cells had the highest LTBP2 expression level and showed higher cellular activity after cisplatin treatment, suggesting that HGC-27 cells expressing high *LTBP2* gene levels have lower cisplatin sensitivity. LTBP2 is a protein affiliated with the fibrillin/LTBP ECM glycoprotein family and plays a vital role in cell adhesion and elastic fiber aggregation (Vehviläinen et al., 2009). The upregulation of EMT levels in GC can usually lead to the development of chemoresistance (Yang et al., 2021). Our previous study also directly confirmed that LTBP2 plays a role in promoting EMT in GC cells (Wang et al., 2018). These results indicate a possible regulatory relationship between abnormal upregulation of *LTBP2* and chemotherapy resistance. This is consistent with the results of this study, which showed that high expression of LTBP2 mediated high cisplatin resistance. Furthermore, higher levels of LTBP2 expression were observed in the HGC-27 cisplatin-resistant cell line. Unexpectedly, the sensitivity of both wild-type HGC-27 and HGC-27/DDPR cells to cisplatin was enhanced after silencing the *LTBP2* gene, and the proliferation activity of the cells was significantly inhibited, implying that silencing *LTBP2* reduced the cisplatin resistance of HGC-27 and HGC-27/DDPR cells.

Analysis of the correlation between *LTBP2* and the *NF-κB2* gene using data from shared clinical samples of gastric cancer patients showed a significant positive correlation. Silencing of *LTBP2* significantly suppressed the regulation of the *NF-κB2* gene and protein expression, which belongs to the NF-κB nonclassical transcriptional pathway and exerts transcriptional regulation in the translocated nucleus through the combination of the functional protein subunit p52 and Bcl-3 (Pan et al., 2022). Immunofluorescence assays further confirmed strong positive expression of the p52 protein in the nucleus of HGC-27/DDPR cells with cisplatin resistance. In contrast, the expression of NF-κB2 p52 in the nucleus was significantly suppressed after silencing *LTBP2*. The protein expression levels of Bcl-3 and cyclin D1, the downstream transcriptional regulatory proteins of NF-κB p52, were also considerably suppressed after the silencing of *LTBP2* (Zhang et al., 2007).

Bcl-3 binding to the p52 complex strongly activates the cyclin D1 promoter and can bind to NF-κB, the proximal site of the cyclin D1 promoter. Disorder of Bcl-3 may upregulate cancer cell proliferation levels by upregulating cyclin D1 and stimulating tumors to undergo G1 phase transition (Westerheide et al., 2001). This finding suggests that silencing *LTBP2* has a repressive effect on NF-κB2 p52 protein transcription, affecting the regulation of cell proliferation capacity by downstream pathways. Increased transcriptional regulation of cyclin D1 effectively increases chemoresistance and stimulates gastric cancer cell proliferation capacity (Jiang et al., 2018; Roliński et al., 2021). In our study, re-expression of the *NF-κB2* gene in HGC-27 and HGC-27/DDPR cells, in which *LTBP2* had already been silenced, effectively reversed the increased effect of silencing *LTBP2* on cisplatin sensitivity in GC cells and significantly upregulated the protein expression levels of p52, Bcl-3, and cyclin D1.

In conclusion, we demonstrated that silencing LTBP2 significantly reduced cisplatin resistance and inhibited the proliferation ability of GC cells by silencing *LTBP2* in wild-type and cisplatin-resistant HGC-27 cells in this study. Mechanistically, silencing *LTBP2* may inhibit cell proliferation and cisplatin resistance by suppressing the NF-κB nonclassical pathway: p52/Bcl3/cyclin D1. The present study provides a more experimental basis for the study and target selection of LTBP2 in GC. This study provides a new direction for clinically targeted molecular therapy and treatment for patients with cisplatin-resistant GC.
